# Knowledge mobilization for and with people with lived experience of dementia

**DOI:** 10.1002/alz70858_100225

**Published:** 2025-12-25

**Authors:** Jennifer Bethell, Carrie A McAiney, Juanita‐Dawne R Bacsu, Engagement of People with Lived Experience of Dementia Advisory Group

**Affiliations:** ^1^ KITE Research Institute, Toronto Rehabilitation Institute ‐ University Health Network, Toronto, ON, Canada; ^2^ University of Waterloo, Waterloo, ON, Canada; ^3^ Schlegel‐UW Research Institute for Aging, Waterloo, ON, Canada; ^4^ Thompson Rivers University, Kamloops, BC, Canada

## Abstract

**Background:**

Engaging people with lived experience of dementia (i.e., people living with dementia, family/friend care partners) in all phases of research contributes to more relevant and meaningful research findings. Mobilizing knowledge to diverse audiences is critical to getting scientific findings into the hands of knowledge users, including people with lived experience of dementia. To ensure the goals of patient engagement and knowledge mobilization (KM) were achieved, two cross‐cutting programs within the Canadian Consortium on Neurodegeneration in Aging (CCNA) were established: a KM program to support KM activities and a lived experience program, Engagement of People with Lived Experience of Dementia (EPLED). The EPLED Advisory Group consists of people with lived experience of dementia, to support the engagement of people with lived experience in research.

**Method:**

In addition to their respective core activities, the KM and EPLED programs united to work collaboratively to achieve shared goals of increasing the involvement of people with lived experience throughout the CCNA network, including in KM. Together, the programs undertook activities to raise the profile of people with lived experience of dementia, involving EPLED in KM activities as well as planning and decision making. Over time, this led to a culture shift within CCNA where researchers increasingly sought out and involved the perspectives of people with lived experience in research and KM activities.

**Result:**

People with lived experience of dementia are now key collaborators at all stages of scientific research within CCNA, including in planning and executing KM activities. Evaluation data indicate these activities are valued by researcher, trainee and public audiences.

**Conclusion:**

The integration of people with lived experience of dementia within CCNA's research and KM projects has been a resounding success. EPLED and KM will continue to collaborate on integrating people with lived experience in CCNA's research activities, and support researchers in developing KM activities and skills.

